# Molecular Screening of *Sarcocystis* spp. in Grazing Sheep (*Ovis aries*) and Shepherd Dogs (*Canis lupus familiaris*) from Central Portugal

**DOI:** 10.3390/ani15233479

**Published:** 2025-12-02

**Authors:** Sara Gomes-Gonçalves, Ricardo J. Figueiredo, Soraia Rodrigues, Jaqueline T. Bento, Sérgio Santos-Silva, Daniela Almeida, Rita Cruz, Fernando Esteves, Alexandra Lameira Baptista, Maria Aires Pereira, Luís Cardoso, João R. Mesquita

**Affiliations:** 1 School of Medicine and Biomedical Sciences (ICBAS), Universidade do Porto (UP), 4050-313 Porto, Portugal; 2 Escola Superior Agrária de Viseu, Instituto Politécnico de Viseu, 3504-510 Viseu, Portugalalexabaptista@esav.ipv.pt (A.L.B.);; 3 Epidemiology Research Unit (EPIUnit), Instituto de Saúde Pública da Universidade do Porto, 4050-091 Porto, Portugal; 4 Laboratório para a Investigação Integrativa e Translacional em Saúde Populacional (ITR), 4050-600 Porto, Portugal; 5 CERNAS-IPV Research Centre, Instituto Politécnico de Viseu, 3504-510 Viseu, Portugal; 6 Global Health and Tropical Medicine (GHTM), Associate Laboratory in Translation and Innovation Towards Global Health (LA-REAL), Instituto de Higiene e Medicina Tropical (IHMT), Universidade NOVA de Lisboa (UNL), 1349-008 Lisboa, Portugal; 7 Department of Veterinary Sciences, Universidade de Trás-os-Montes e Alto Douro (UTAD), Quinta de Prados, 5000-801 Vila Real, Portugal; lcardoso@utad.pt; 8 Department of Veterinary Sciences and Animal and Veterinary Research Centre (CECAV), Universidade de Trás-os-Montes e Alto Douro (UTAD), Quinta de Prados, 5000-801 Vila Real, Portugal; 9 Associate Laboratory for Animal and Veterinary Science (AL4AnimalS), 1300-477 Lisboa, Portugal; 10 Centro de Estudos de Ciência Animal (CECA), Instituto de Ciências, Tecnologias e Agroambiente (ICETA), Universidade do Porto (UP), 4051-401 Porto, Portugal

**Keywords:** Portugal, *Sarcocystis* spp., sheep, shepherd dogs

## Abstract

**Simple Summary:**

Sheep can be infected by parasites of the genus *Sarcocystis*, which form cysts in muscles and internal organs and may cause disease, economic losses, and animal welfare concerns. These protozoa have an obligate two-host life cycle, requiring intermediate hosts such as sheep and definitive hosts, including dogs or wild carnivores. Infections can be subclinical but may also cause weight loss, anemia, neurological signs, abortion, or death, depending on species and host susceptibility. In Portugal, where sheep farming is a key agricultural sector, data on the prevalence of *Sarcocystis* spp. in meat-producing animals were lacking. This study analyzed brain and blood samples from sheep and stool samples from shepherd dogs to detect the presence of the parasite using molecular methods. *Sarcocystis* sp. was identified in 4.9% of brain tissue samples, while no DNA was detected in blood or dog stool samples, representing the first molecular confirmation in Portuguese sheep destined for human consumption. Although dogs tested negative, other definitive hosts, including wild carnivores, may contribute to parasite circulation. These findings highlight the importance of understanding *Sarcocystis* distribution and transmission dynamics to support animal health, minimize economic losses, and guide effective control strategies in Portuguese sheep farming.

**Abstract:**

*Sarcocystis* spp. are cyst-forming protozoan parasites with a global distribution that infect a wide range of domestic and wild animals, impacting both animal health and livestock productivity. In sheep, infections can cause clinical disease, reproductive losses, and economic damage, particularly when pathogenic species such as *Sarcocystis tenella* are involved. Grazing sheep, including breeds such as the Serra da Estrela from central Portugal, are at increased risk due to frequent contact with shepherd dogs, which serve as definitive hosts. Despite their significance, data on the occurrence and distribution of *Sarcocystis* spp. in Portuguese sheep remain limited. This study analyzed 179 samples collected in central Portugal during 2024, including 41 brain tissues and 88 blood samples from sheep, and 50 stool samples from shepherd dogs, using conventional PCR and bidirectional Sanger sequencing. *Sarcocystis* sp. closely related to *S. tenella* was detected exclusively in sheep brain tissue, with a prevalence of 4.9% (2/41; 95% CI: 0.60–16.53), while no parasite DNA was found in blood or dog samples. These results provide the first molecular confirmation of *Sarcocystis* spp. closely related to *S. tenella* in Portuguese sheep raised for human consumption and establish baseline data for future epidemiological surveillance and control strategies.

## 1. Introduction

*Sarcocystis* spp. are among the most common cyst-forming protozoan parasites with worldwide distribution in various hosts [1]. Infection in ruminants not only raises veterinary health concerns but also results in considerable economic losses in the livestock industry. Data from a previous study conducted in Spain estimated the annual losses of approximately 20 million EUR, mostly due to carcass condemnation, reduced meat quality, and decreased wool and milk production [2,3,4]. In Portugal, the sheep and goat meat production industry represents an important agricultural sector, ranking among the top 10 producers in Europe according to Eurostat, with 9.18 thousand tons of meat from slaughterhouses in 2024. This circumstance further highlights the economic importance of this industry for the country and its contribution to national food security [5].

*Sarcocystis* spp. are parasites belonging to the phylum Apicomplexa and have an obligatory heteroxenous life cycle depending on a prey–predator relationship between definitive hosts (predators, such as canids and felids) and intermediate hosts (prey, including cattle, sheep, horses, goats, and pigs) [6]. Currently, more than 200 species of the genus *Sarcocystis* have been recorded in different animals [7]. Infection occurs when intermediate hosts consume food or water contaminated with oocysts or free sporocysts, whereas definitive hosts, such as predators or scavengers, become infected through the ingestion of animal tissues containing mature sarcocysts [8,9,10].

Sheep can harbor at least six species, namely *Sarcocystis arieticanis*, *Sarcocystis gigantea*, *Sarcocystis medusiformis*, *Sarcocystis microps*, *Sarcocystis mihoensis* and *Sarcocystis tenella*. Among them, *S. tenella* and *S. arieticanis* are considered pathogenic, with the latter slightly less virulent [11]. Clinical signs of infection by *S. tenella* results in anorexia, weight loss, fever, anemia, hair loss, abortion, premature birth, neurologic signs, myositis, and death [9].

The importance of canids in the transmission of *Sarcocystis* spp. is broadly known. The use of shepherd dogs to guard flocks is a common practice in Portuguese sheep farming, which, unlike companion dogs, are in closer contact with flocks, increasing the likelihood of contact to sheep. *Sarcocystis* spp. cause little harm to their carnivore definitive host since infection is usually subclinical or, less commonly, resulting in mild diarrhea [12]. In contrast, herbivorous intermediate hosts often experience severe tissue damage, leading to higher mortality and considerable economic losses [12,13].

In Portugal, the circulation of *Sarcocystis* spp. has been recorded in wild boars for human consumption as well as in dogs and stray cats [14,15,16,17]. However, when focusing on the impact on livestock industry in Portugal, data on the occurrence of *Sarcocystis* spp. in sheep intended for human consumption is currently lacking. This study aims to address this gap by performing molecular detection of *Sarcocystis* spp. in sheep brain tissue and blood, as well as in stool samples from shepherd dogs, to better understand the occurrence and transmission dynamics of these parasites.

## 2. Material and Methods

### 2.1. Sampling

**Figure 1:**
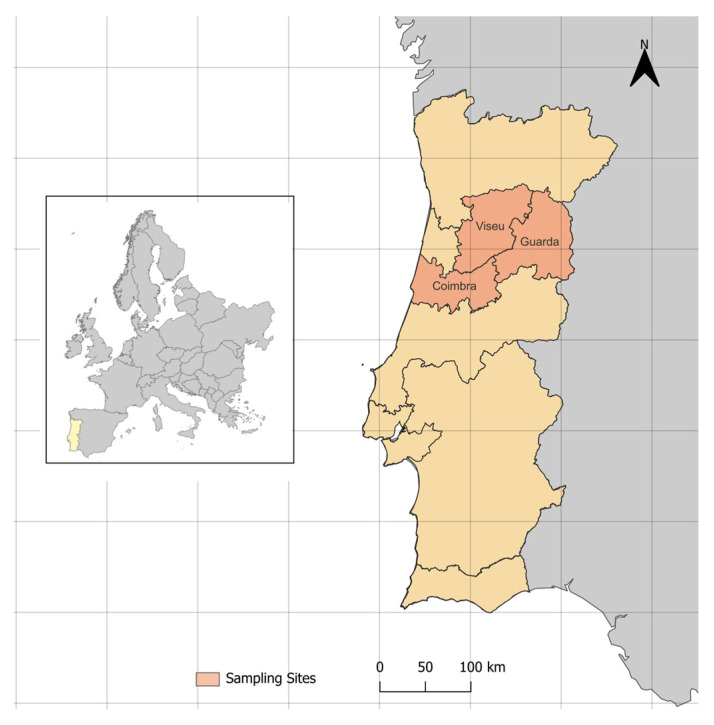
Geographic representation of the sampling areas included in the study (orange shading). All other Portuguese administrative regions are shown in light yellow. The inset map shows Portugal’s location within Europe, with Portugal highlighted in yellow. A north arrow and scale bar are included to indicate orientation and distance.

A total of 179 samples were collected during 2024, comprising 41 brain tissue samples and 88 blood samples from different sheep, as well as 50 stool samples from shepherd dogs. Brain tissue samples were obtained from sheep originating from the central region of Portugal (Figure 1) during routine slaughter procedures for meat production in March 2024. This approach allowed the use of material that was readily available and would otherwise have been discarded, avoiding additional handling or invasive procedures on animals specifically for this study. Blood samples were collected from clinically healthy female sheep as part of the national brucellosis control program. These samples represented 16 distinct farms within the districts of Guarda, Viseu, and Coimbra, with herd sizes ranging from 27 to 700 animals, reflecting the diversity of sheep production systems in the region. Residual material from 5 mL of blood collected in EDTA tubes via jugular venipuncture was used for molecular analyses, ensuring no additional invasive procedures were performed.

Stool samples were collected from 50 shepherd dogs in the same geographical regions to evaluate their potential role as definitive hosts in the *Sarcocystis* life cycle. The sampled dogs comprised 20 females and 30 males of various breeds, including Border Collie, Gado Transmontano, Serra Aires, Serra da Estrela, as well as mixed or undefined breeds. All animals were clinically healthy and exhibited no signs of diarrhea at the time of sampling. These dogs were responsible for managing flocks ranging from 3 to 700 sheep, with an estimated average ratio of approximately one dog per 79 sheep, reflecting typical herd-guarding practices in the region. Stool samples were collected immediately after defecation to preserve sample quality and prevent environmental contamination. All samples were transported to the laboratory on the same day under refrigeration at 4 °C and subsequently stored at −20 °C until processing the following day, thereby minimizing potential DNA degradation and ensuring reliable molecular analyses.

Animal handling and sample collection were conducted under the authorizations issued by the Animal Welfare Body of Escola Superior Agrária de Viseu (ORBEA-ESAV), in full compliance with relevant ethical and legal regulations (license no. 03.01/ORBEA/2024; approved on 22 March 2024).

### 2.2. DNA Extraction

Genomic DNA was extracted from 100 mg of sheep brain tissue using a modified protocol of the QIAamp^®^ DNA Mini Kit (Qiagen, Valencia, CA, USA) to optimize both yield and purity. Each tissue sample was incubated with 420 µL of lysis buffer and 25 µL of proteinase K in 2.0 mL Eppendorf tubes, vortexed for 30 s, and centrifuged at 6000× *g* for 2 min. Complete tissue digestion was achieved through incubation at 57 °C for 15 min. Subsequently, 350 µL of the supernatant was transferred to a fresh tube and combined with an equal volume of RTL buffer, vortexed, and briefly centrifuged before proceeding with automated extraction on the QIAcube^®^ platform (Qiagen GmbH, Hilden, Germany) in accordance with the manufacturer’s instructions.

Blood-derived DNA was isolated from 200 µL aliquots of homogenized EDTA-anticoagulated whole blood using the PurePrep96 magnetic bead system (MolGen^®^, Veenendaal, The Netherlands) in conjunction with the BioExtract^®^ SuperBall^®^ kit. This approach ensured high-throughput, reproducible recovery of nucleic acids while minimizing the risk of contamination.

For dog stool samples, fecal material was initially diluted to a 10% suspension in phosphate-buffered saline (PBS, pH 7.2), thoroughly vortexed, and centrifuged at 8000× *g* for 5 min (Eppendorf, Hamburg, Germany) to remove particulate matter. A 140 µL aliquot of the clarified supernatant was subjected to DNA extraction using the QIAamp^®^ DNA Mini Kit on the QIAcube^®^ platform, following the manufacturer’s recommended protocol. Each extraction batch included a non-template control containing RNase-free water and positive 18S rRNA controls to monitor extraction efficiency and detect potential contamination. All purified DNA was stored at −80 °C in RNase-free water until downstream molecular analyses, preserving integrity and ensuring reproducibility across assays.

### 2.3. Molecular Detection of Sarcocystis spp.

Molecular detection of *Sarcocystis* spp. was carried out by conventional PCR in a total reaction volume of 25 µL, using the primer pair Sar-F1 (5′-GCACTTGATGAATTCTGGCA-3′) and Sar-R1 (5′-CACCACCCATAGAATCAAG-3′), which amplifies a fragment of the 18S rRNA gene [18,19]. Reactions were prepared using the Speedy Supreme NZYTaq 2× Green Master Mix (NZYTech, Lisbon, Portugal), which contains DNA polymerase, buffer, dNTPs, and Mg^2+^, facilitating consistent and reproducible amplification. A no-template control, in which RNase-free water was added in place of DNA, was included in each run to monitor for contamination and confirm the specificity of the amplification. All PCR reactions were conducted on a T100 thermocycler (Bio-Rad, Hercules, CA, USA).

The thermal cycling protocol consisted of an initial denaturation at 95 °C for 5 min to ensure complete separation of template DNA strands, followed by 40 cycles of denaturation at 94 °C for 2 s, annealing at 55 °C for 5 s, and extension at 72 °C for 5 s. These short cycle durations correspond to a fast PCR protocol designed to maintain amplification efficiency while minimizing overall run time. A final extension at 72 °C for 10 min was included to ensure complete synthesis of all PCR products, particularly any amplicons that may have been partially extended during the rapid cycles.

Following amplification, PCR products were separated on 1.5% agarose gels prepared with Xpert Green Safe DNA gel dye (GRiSP^®^, Porto, Portugal) and electrophoresed at 120 V for 25 min. This gel concentration allows clear resolution of the expected amplicon size, and staining with the DNA-intercalating dye enables direct visualization under UV illumination.

### 2.4. Sequencing and Phylogenetic Analysis

Amplicons of the expected size were purified using the GRS PCR and Gel Band Purification Kit (GRiSP) and subsequently sequenced in both directions using Sanger sequencing to ensure high-quality bidirectional reads. The resulting sequences were carefully edited, aligned, and analyzed using BioEdit Sequence Alignment Editor (v7.2.5), allowing for the generation of accurate consensus sequences. These consensus sequences were compared against the NCBI GenBank database using BLASTn (https://blast.ncbi.nlm.nih.gov/Blast.cgi, accessed on 22 September 2025) to confirm species identity. *Sarcocystis*-positive sequences were deposited in GenBank under accession numbers PX309295 and PX309296 to enable public access and future comparative studies. For phylogenetic analysis, 15 reference sequences were retrieved from GenBank and aligned using MAFFT v7.490 with the L-INS-i algorithm, which improves alignment accuracy for sequences with variable regions. A maximum likelihood tree was then constructed using IQ-TREE, applying 1000 bootstrap replicates and the best-fit substitution model K3Pu+F+I+R3 to evaluate the robustness of inferred relationships. *Toxoplasma gondii* was included as an outgroup to root the tree and provide a reference for divergence. Tree annotation, visualization, and final figure preparation were performed using the Interactive Tree of Life (iTOL) platform v7, facilitating clear representation of evolutionary relationships among the detected and reference *Sarcocystis* sequences.

### 2.5. Statistical Analysis

The occurrence of *Sarcocystis* spp. was determined by calculating the proportion of positive samples relative to the total number of samples analyzed, with corresponding 95% confidence intervals (95% CI) to quantify the precision of the estimates. Differences in detection rates between sample types, including brain tissue, blood, and stool samples, were evaluated using Fisher’s exact test, which is appropriate for categorical data with small sample sizes or low expected counts. Data processing, including organization, cleaning, and verification of raw datasets, was performed in Microsoft Excel^®^ for Microsoft 365 MSO (Version 2312, Build 16.0.17126.20132, 64-bit). Subsequent statistical analyses, including the calculation of prevalence, confidence intervals, and Fisher’s exact test, were conducted in RStudio version 4.4.2 (Boston, MA, USA) using standard packages for data handling and statistical testing. All analyses were performed with rigorous attention to accuracy, ensuring that both the prevalence estimates and the results of the significance tests reliably reflected the observed data. This approach provided a robust quantitative framework for assessing the presence and distribution of *Sarcocystis* spp. across the different sample types included in the study.

## 3. Results

A total of 41 sheep brain tissue samples, 88 sheep blood samples, and 50 shepherd dog stool samples were screened for *Sarcocystis* spp. *Sarcocystis* sp. was detected exclusively in brain tissue, with an occurrence of 4.9% (2/41; 95% CI: 0.60–16.53). Fisher’s exact test did not reveal statistically significant differences among the different sample types (*p* > 0.05). No *Sarcocystis* DNA was detected in sheep blood or in dog stool samples. The sequences obtained from the positive brain tissue samples revealed 100% identity with *S. tenella*, and the top 10 highest hits from the BLAST analysis are summarized in Table 1. These results provide precise molecular confirmation of closely related *S. tenella* in the sheep brain samples analyzed and establish a baseline for further epidemiological investigation in Portuguese livestock.

**Figure 2:**
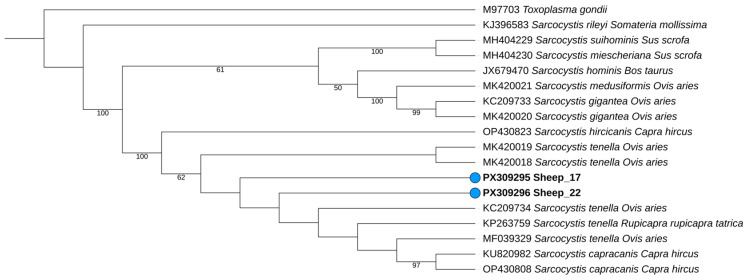
Phylogenetic tree generated using IQ-TREE software v2.3.6, incorporating reference *Sarcocystis* sequences obtained from the GenBank database. Sequences obtained are highlighted with blue circles and bold text. Reference sequences are presented without special formatting and include their accession numbers, *Sarcocystis* spp. and host. The analysis was conducted under the K3Pu+F+I+R3 of nucleotide substitution, with 1000 bootstrap replicates to assess branch support. Only bootstrap values ≥ 60% are shown. Tree annotation and visualization were performed using the Interactive Tree of Life (iTOL) platform version 7.

Phylogenetic analysis, performed using reference sequences retrieved from GenBank, showed that both sheep-derived sequences clustered within a well-supported *S. tenella* clade as displayed in Figure 2**,** indicating that *Sarcocystis* sp. is closely related to *S. tenella*. The sequences obtained in this study, highlighted in bold and marked with blue circles, clustered with a 62% bootstrap value, which provides moderate support for the stability of this grouping.

## 4. Discussion

In this study, *Sarcocystis* sp. closely related to *S. tenella* was detected in sheep brain tissue, with a prevalence of 4.9%. This observation represents the first molecular confirmation of ovine sarcocystosis in animals raised for human consumption in Portugal and fills an important gap in the epidemiological knowledge of this parasite within the country. Reports of *Sarcocystis* spp. in Portuguese sheep are otherwise scarce, making these findings particularly interesting. Comparable studies in other countries have documented variable prevalence rates in brain tissue, ranging from 1.25% in Iran [20] to 52.51% in China [21], while a case report in the United Kingdom described the parasite in cerebrospinal fluid [22]. Beyond brain tissue, *Sarcocystis* spp. have been detected in multiple organs of sheep in diverse geographic regions, including Spain [4], Egypt [23], Lithuania [24], Brazil [25], Malaysia [26], Iraq [27], Japan [28] and Tunisia [29]. This wide distribution highlights the parasite’s ability to infect different tissues and adapt to varied environmental conditions and host populations. The current findings underscore the importance of systematic surveillance to determine the prevalence, tissue tropism, and potential economic and animal health impacts of *Sarcocystis* spp. in Portuguese livestock, providing a basis for the development of effective monitoring and control strategies in sheep farming systems.

Although *Sarcocystis tenella* is not generally considered zoonotic, its presence in sheep has important veterinary and economic implications. Data on the economic impact of *Sarcocystis* spp. infection in Portuguese sheep are currently lacking, but studies in neighboring Spain estimate losses of approximately 20 million EUR annually, primarily due to carcass condemnation, reduced meat quality, and decreased wool and milk production [4]. Pathogenic species transmitted by canids, including *S. tenella* and *S. arieticanis*, can have severe consequences for animal health. Primary infection during gestation may result in fetal death, abortion, or premature lambing [30]. In addition, infected animals may exhibit neurological signs, acute disease with high mortality in young lambs, or chronic disease in older animals. Infection can also impair growth rates and production efficiency, negatively affecting wool and milk yield [2,31,32].These outcomes underscore the importance of monitoring and controlling *Sarcocystis* spp. infections to mitigate economic losses and safeguard animal welfare in sheep production systems.

Despite the fact that *Sarcocystis* spp. are more commonly detected in tissue samples, blood was also examined, as *Sarcocystis* undergoes a brief phase of circulation in the bloodstream of the intermediate host before invading muscle cells to form tissue cysts [33]. Detecting the parasite in blood would, in principle, enable the diagnosis of initial infection in living animals rather than relying solely on post-mortem tissue analysis or, less likely, on biopsies [34]. However, all blood samples in the present study tested negative. Comparable results were recently reported [35], who also failed to detect *Sarcocystis* DNA in sheep blood. Taken together, these findings indicate that detection of *Sarcocystis* spp. in ovine blood is difficult, and blood may therefore have limited value as a diagnostic sample for ovine sarcocystosis.

*Sarcocystis* spp. use canids as definitive hosts, shedding sporocysts in feces that can infect sheep [30]. On many farms, dogs are kept to guard flocks, creating frequent opportunities for sheep to come into contact with dog feces. This interaction can increase the risk of transmission, as sheep may ingest sporocysts from contaminated pastures, feed, or water. Dogs’ defecation behavior near sheep housing or feeding areas can further facilitate environmental contamination, sustaining the parasites’ life cycle on farms [24]. To investigate this potential link, stool samples from 50 shepherd dogs in the study area were analyzed, but all tested negative for *Sarcocystis*. A previous survey on dogs in Portugal reported a prevalence of only 0.3%, suggesting that infections in domestic dogs are uncommon [15]. Although close contact could in fact increase transmission risk, the absence of infection in these animals indicates that they may not be a major source of infection in this region. Other definitive hosts, particularly wild canids such as wolves (*Canis lupus*) and foxes (*Vulpes vulpes*), may therefore play a more significant role, as the presence of *Sarcocystis* in these hosts has been previously reported in Portugal [31], highlighting the need for further studies that include not only domestic but also wildlife hosts.

Despite the results obtained, this study has several important limitations. The sample size was modest, which may limit both the accuracy and the representativeness of the findings. Sampling was cross-sectional and restricted to a single region and season, which may have influenced detection by missing temporal and geographic variation as well as periods of peak shedding or infection.

Only one tissue sample per animal was analyzed, which may have underestimated the true prevalence of infection. In addition, DNA was extracted from whole tissue rather than isolated cysts, as a result, species-level identification remains uncertain and possible coinfection cannot be ruled out. Species identification in this genus is strengthened when ultrastructural features of the cyst wall are available, typically through transmission electron microscopy or detailed histology. Although such analyses were not part of the present work, the genetic data still provide meaningful insight into the diversity of the detected taxa. Future research should integrate both molecular and ultrastructural approaches for a more comprehensive characterization.

Although 50 shepherd dogs were tested and all were negative, intermittent shedding and low prevalence among domestic dogs can reduce the probability of detection, and negative stool PCR results do not entirely exclude a potential role for domestic dogs in local transmission.

Detection of *Sarcocystis* from fecal material is challenging. General primers, as used in this study, may not be sufficient to reliably detect the parasite due to short DNA fragments, limited DNA quantity, and potential DNA degradation caused by freezing. Implementing nested or multiplex qPCR assays with internal controls would improve reliability by identifying possible inhibitors. Future studies should consider reviewing optimal methods for detecting the sexual phase of *Sarcocystis* and combining morphological and molecular approaches to strengthen detection reliability.

The absence of correlation between serological and fecal findings limits the ability to confirm active parasite shedding. Future studies incorporating sero-positive dogs and analyzing their fecal samples would provide a more targeted and sensitive approach, thereby strengthening epidemiological conclusions.

The reliance on a single marker (18S rRNA) limits species-level resolution, and future studies should incorporate additional molecular markers such as 28S rRNA, coxI, or ITS1, as well as transmission electron microscopy or ultrastructural analysis of the cyst wall to confirm species identity. Phylogenetic analysis in this study yielded a bootstrap support of 62%, indicating moderate confidence. The limited discriminatory power of 18S rRNA means that closely related species, including *S. tenella*, *S. arieticanis*, and *S. capracanis*, may not be fully resolved. Although *S. microps* and *S. mihoensis* have been reported in sheep [11], their sequences are unavailable in databases, so potential overlap with these taxa cannot be excluded. Future studies should incorporate additional molecular markers such as 28S rRNA, coxI, or ITS1. Consequently, caution is warranted when interpreting species assignments based solely on this marker. Moreover, to fully assess tissue tropism and prevalence, future research should include skeletal and cardiac muscles in addition to the current tissue samples. Finally, the lack of histopathology data on positive sheep limits interpretation of pathogenicity.

## 5. Conclusions

This study provides the first molecular evidence of *Sarcocystis* spp. closely related to *S. tenella* in Portuguese sheep raised for human consumption, with a prevalence of 4.9% detected in brain tissue samples. The detection was limited to brain tissue, while no parasite DNA was identified in peripheral blood or in stool samples from domestic shepherd dogs. Although dogs tested negative in this study, their role as definitive hosts in the transmission cycle cannot be completely excluded, particularly given the well-established involvement of canids in the life cycle of *Sarcocystis*. Additionally, the potential contribution of wild canids or other carnivorous species to parasite circulation warrants further investigation to fully characterize the ecological and epidemiological context of infection.

Given the documented veterinary and economic consequences of *S. tenella* infection, which include reproductive losses, decreased growth rates, and overall reduced productivity, there is a clear need for more comprehensive epidemiological studies. Future investigations should encompass larger sample sizes, inclusion of multiple tissue types beyond brain and blood, seasonal sampling to account for temporal variation, and systematic examination of both domestic and wildlife definitive hosts. Expanding the scope of research in this manner will provide a more complete understanding of the prevalence, tissue tropism, and transmission dynamics of *Sarcocystis* spp. in Portugal. Ultimately, such data are essential for informing surveillance programs, guiding risk assessments, and developing effective control strategies to safeguard animal health and minimize economic losses in the sheep industry.

## Figures and Tables

**Table 1:** Results of the BLAST analysis for *Sarcocystis* spp.

Accession Number	Query Cover (%)	E-Value	Percentage Identity (%)	Specie	Location
MW832474	100	0.0	100	*Sarcocystis tenella*	Spain
MW832473	100	0.0	100	*Sarcocystis tenella*	Spain
MG515216	100	0.0	100	*Sarcocystis tenella*	Egypt
MW832475	100	0.0	100	*Sarcocystis tenella*	Spain
KP263759	100	0.0	100	*Sarcocystis tenella*	Poland
KR155209	100	8 × 10^−80^	100	*Sarcocystis* sp.	Malaysia
KR155230	100	8 × 10^−80^	100	*Sarcocystis* sp.	Malaysia
KP263752	100	8 × 10^−80^	100	*Sarcocystis tenella*	Poland
MW832472	100	8 × 10^−80^	100	*Sarcocystis tenella*	Spain
MT569891	100	8 × 10^−80^	100	*Sarcocystis tenella*	Iran

## Data Availability

No datasets were generated or analyzed during the current study.
